# Mechanism of Yifei Decoction Combined with MitoQ on Inhibition of TGF*β*1/NOX4 and PDGF/ROCK Signal Pathway in Idiopathic Pulmonary Fibrosis

**DOI:** 10.1155/2021/6615615

**Published:** 2021-05-28

**Authors:** Lijuan Chen, Chengzhong Lan, Hong Xiao, Xiaoli Zhang, Xiangrong Qi, Li Ouyang, Yanbin Yang, Fengying Wang, Qihua Jin, Yi Sun

**Affiliations:** Yunnan Provincial Hospital of Traditional Chinese Medicine, Kunmin, Yunnan 650000, China

## Abstract

**Background:**

Rho-related coiled helix forming protein kinase (Rho-ROCK) and another important fibrogenic factor-PDGF play a critical role in collagen deposition in rat lung tissue. Yifei decoction (YFT), a Chinese herbal decoction, has been used to treat idiopathic pulmonary fibrosis (IPF) in clinical practice and has produced positive outcomes; however, convincing evidence is currently lacking. The present study aimed to investigate the effects of YFT combined with MitoQ in rats with IPF and to explore the underlying mechanism.

**Methods:**

Rat IPF model was established by endotracheal injection of 5 mg/kg BleomycinA5 into the specific pathogen-free SD rats. MitoQ (6.5 *μ*mol/kg once daily), YFT (10 ml/kg once daily), and MitoQ + YFT (6.5 *μ*mol/kg + 10 ml/kg once daily) were used to treat the rat model for 4 weeks, respectively. The normal rats without IPF were used as the controls. After 4 weeks of drug treatment, lung histopathology was assessed. Immunohistochemistry was used to detect the expression of fibronectin and collagen IV in lung tissue. The expression of IL-6, IL-1*β*, TNF-*α*, GSH-Px, SOD, MDA, and hydroxyproline was determined by enzyme-linked immunosorbent assay. The expressions of TGF*β*1, NOX4, PDGFR-*β*, and ROCK1 were determined using real-time quantitative PCR and Western blot.

**Results:**

After 4 weeks of drug treatment, comparison of the MitoQ + YFT group with the IPF group showed that lung injury scores, W/D, lung tissue hydroxyproline, fibronectin, collagen IV content, and IL-6, IL-1*β*, TNF-*α*, and MDA levels were significantly lower (*P* < 0.05), as well as the expression of TGF*β*1, NOX4, PDGFR-*β*, and ROCK1, but the activity of GSH-Px and SOD was higher (*P* < 0.05).

**Conclusion:**

MitoQ combined with YFT can improve lung injury in rats with pulmonary fibrosis by reducing the secretion of proinflammatory cytokines and inhibiting TGF*β*1/NOX4 and PDGF/ROCK signaling pathways. It may provide a new method for the treatment of pulmonary fibrosis.

## 1. Introduction

Idiopathic pulmonary fibrosis (IPF) is a chronic progressive idiopathic interstitial pneumonia (IIP). IPF causes irreversible damage to lung function, which leads to respiratory failure, among which middle-aged and elderly men have a high incidence [1]. According to worldwide assessment, the annual incidence of IPF in North America and Europe is 2.8–9.3 per 100000 people. The number of cases per 100000 people in Asia and South America is 2.23–10. As the incidence of IPF is related to environmental factors and occupational exposure, different regions lead to different incidences of IPF [2]. The pathological manifestations of IPF are the destruction or collapse of alveolar structure and its epithelial cells, the activation and proliferation of pulmonary fibroblasts, and the excessive accumulation of extracellular matrix, which lead to the increase of pulmonary interstitial and the decrease of pulmonary function [3]. The pathogenesis of pulmonary fibrosis is currently unclear. In recent years, many studies have shown that TGF-*β*/Smad promotes the transformation of myofibroblasts in the process of pulmonary fibrosis, which constitutes a relatively clear mechanism in the pathogenesis of organ fibrosis [4]. The specific expression of *α*-SMA in myofibroblasts and Rho-ROCK signal pathways can regulate the expression of *α*-SMA through a complex phosphorylation/dephosphorylation cascade. These studies have demonstrated that Rho-related coiled helix forming protein kinase (Rho-ROCK) and another important fibrogenic factor-PDGF play a key role in collagen deposition in rat lung tissue [5].

In recent years, with the understanding of the pathogenesis of pulmonary fibrosis by traditional Chinese medicine, some progress has been made in the treatment of pulmonary fibrosis with traditional Chinese medicine [6–8]. Yifei decoction is a Chinese herbal medicine composed of *Cortex Mori, Atractylodes macrocephala* Koidz.*, Fructus Aurantii Immaturus, peach kernel, Salvia miltiorrhiza, Platycodon grandiflorus*, and *licorice* [9]. Several active components of Yifei decoction have been proved to be bioactive *in vivo*. *Cortex Mori* can prevent diabetic nephropathy by inhibiting inflammation and fibrosis in the rat model [10]. *Atractylodes macrocephala* polysaccharide enhances the secretion of cytokines by stimulating TLR4-MyD88-NF- *κ*B signal pathway in mouse spleen [11]. *Peach kernel* oil downregulates the expression of tissue factor in ApoE knockout mice and reduces atherosclerosis [12]. *Licorice* components involved in CYP450 and Nrf2 pathways have detoxification and antioxidation in paraquat-induced acute lung injury in mice [13]. In the early clinical application of Yifei decoction in the diagnosis and treatment of patients with pulmonary fibrosis, we found that Yifei decoction significantly improved the symptoms of patients with pulmonary impotence, and some experimental studies found that a single traditional Chinese medicine could increase the curative effect of IPF patients [14]. Other studies have reported that Yifei decoction can affect the lungs and kidneys of IPF rats. It is preliminarily confirmed that the expression of PDGF in the lungs of rats decreased after treatment, but its specific mechanism is currently unclear [15]. In addition, Yifei decoction also treats bronchopneumonia, chronic obstructive pulmonary disease, and asthma [16–18]. Considering the properties of these traditional Chinese medicines, and based on the previous research results of our team, this study used Yifei decoction to treat IPF and observe its effect on TGF*β*1/NOX4 and PDGF/ROCK signal pathway.

Mitochondrial coenzyme Q (MitoQ) is a new type of mitochondrial-targeted antioxidants. It is a lipophilic cationic triphenylphosphate ion that binds to ubidecarenone and can target aggregation in mitochondria. Pandecenone is not only a key cofactor in the electron transport chain but also an important cell membrane antioxidant [19]. The mechanism of MitoQ is that ubidecarenone is reduced to pantothenol, which can reduce the oxidative stress of mitochondrial lipid intima, and can also be used as an important lipophilic antioxidant to activate uncoupling proteins, reduce the production of free radicals, and prevent the oxidative modification of proteins, lipids, and DNA by free radicals. In the process of IPF, mitochondria activate the TGF*β*1 pathway after excessive stress during oxidative stress, and NOX4 is overexpressed in NADPH oxidase, which further promotes the process of pulmonary fibrosis [20–23], which may be one of the most critical processes in the process of IPF, which may be effectively reversed by MitoQ.

In this study, we observed the lung injury, collagen content, and TGF*β*1/NOX4 and PDGF/ROCK signal pathway of pulmonary fibrosis rats after the administration of Yifei decoction combined with MitoQ. This study aimed to observe the mechanism by which Yifei decoction combined with MitoQ improves lung injury and inhibiting TGF*β*1/NOX4 and PDGF/ROCK.

## 2. Materials and Methods

### 2.1. Experimental Animals

A total of 30 specific pathogen-free (SPF) Sprague-Dawley (SD) rats, aged 3 months, weighing 230 ± 20 g, were selected. The experimental animals were purchased from Chengdu Dashuo Experimental Animal Co., Ltd. (license no. SYXK 2019-189). The rats were kept in cages with a controlled temperature and light cycle (24°C and 12/12 h light cycle) and were provided with free access to food and water. The humidity was 40%. The present study was approved by the Animal Care Unit and Use Committee of West China Hospital of Sichuan University (No. 20211131A).

### 2.2. Experimental Reagents

Yifei decoction (YFT) was purchased from the pharmacy of Yunnan traditional Chinese Medicine Hospital. Mitochondrial coenzyme Q was purchased from MitoQ, Australia Antipodean Pharmaceuticals; kits for H&E staining from Beyotime Institute of Biotechnology; kits of interleukin-1*β* (IL-1*β*), interleukin-6 (IL-6), and tumor necrosis factor-*α* (TNF-*α*) from Shanghai Zhuocai Biotechnology Co., Ltd.); kits of superoxide dismutase (SOD), malondialdehyde (MDA), and glutathione peroxidase (GSH-Px) from Nanjing Jiancheng Bioengineering Institute; RNA Trizol Reagent from Hefei Bomei Biotechnology Co., Ltd. Experimental primary antibodies (fibronectin (FN), collagen IV (ColIV), transforming growth factor-*β*1 (TGF-*β*1), NADPH oxidase 4 (Nox4), platelet-derived growth factor *ß*-receptor (PDGFR-*β*), and Rho-associated coiled-coil protein kinase 1(ROCK-1)) were purchased from Abcam (Shanghai) Trading Co., Ltd. Secondary antibody (horseradish peroxidation enzyme-labeled goat anti-rabbit antibody) was purchased from Cell Signaling Technology, Inc.

### 2.3. Experimental Instruments

Real-time fluorescence quantitative (RT-PCR) instrument (PIKORed 96, American ThermoFisher), full-function enzyme label instrument MK3 (American ThermoFisher), chemiluminescence gel imager 5200 (Shanghai Tianneng Technology), and the BA210Digital digital trinocular camera micro camera system (McAudi Industry Group) were used.

### 2.4. Preparation of Yifei Decoction

Yifei decoction is composed of *Cortex Mori, Atractylodes macrocephala* Koidz.*, Fructus Aurantii Immaturus, peach kernel, Salvia miltiorrhiza, Platycodon grandiflorus*, and *licorice*. Put the traditional Chinese medicine tablets in an electric boiling pot, soak in mineral water equivalent to 5 times the amount of medicinal materials for 1 hour, boil for 30 minutes, and filter with gauze. Add 6 times the mineral water to the dregs and continue to boil for 20 minutes, and then filter with gauze. The two filtrates were combined, concentrated in vacuum by rotary evaporator, and concentrated and evaporated to extract. Then, put the extract into the freeze dryer, and prepare freeze-dried powder, subpack, and −80°C freeze storage.

### 2.5. Rats Model of Bleomycin-Induced Idiopathic Pulmonary Fibrosis and Medication of Experimental Animals

A total of 30 SPF rats were adaptively fed for 1 week, followed by being randomly divided into 5 groups with 6 animals in each group, namely, the control group (control), model group (IPF), MitoQ group (IPF + MitoQ), Yifei decoction group (IPF + YFT), and MitoQ combined with Yifei decoction group (IPF + MitoQ + YFT). Except for the control animals, all of the animals in the other four groups were anesthetized with pentobarbital sodium (35 mg/kg) and received 5 mg/kg Bleomycin A5 (BLM A5) by endotracheal injection in order to establish an idiopathic pulmonary fibrosis model. Starting from the second day after bleomycin injection, rats in the control group and IPF group were intragastrically administered 0.9% normal saline (2 mL), rats in the IPF + MitoQ group were injected with MitoQ (6.5 *μ*mol/kg) through the tail vein once daily, rats in the IPF + YFT group were intragastrically administered Yifei decoction (10 ml/kg) 1 time per day, and rats in the IPF + MitoQ + YFT group were injected with MitoQ (6.5 *μ*mol/kg) and then treated with Yifei decoction (10 ml/kg) once daily. Each group was given drugs for 4 weeks. The dosage of Yifei decoction and MitoQ refers to the research reports of Zhang et al. [14] and Li et al. [24], respectively.

### 2.6. Sample Collection

After 4 weeks of drug treatment, rats in each group were sacrificed using intraperitoneal injection of anesthetic pentobarbital sodium (35 mg/kg) method. Afterward, the intact lung tissues of rats were sampled and washed with buffer, left lung tissues of rats were measured as the ratio of lung wet/dry (W/D), and left lung tissues of rats were sampled and fixed with 4% formaldehyde solution. Superior lobe and middle lobe of the right lung were, respectively, sampled, washed clean, cut into pieces, and stored at −80°C. All the rats had no pain during the execution and were in accordance with the principles of medical animal ethics.

### 2.7. Hematoxylin and Eosin (H&E) Staining

Left lung tissues having been fixed with 4% formaldehyde solution were embedded in paraffin. Before staining, the sections were incubated at 60°C for 1 h, dewaxed with xylene, and rehydrated through a series of ethanol solutions. The embedded wax blocks were sliced by a slicer with a thickness of 3–5 *μ*m. Finally, the sections of lung tissues were stained with H&E. The BA200 Digital trinocular camera micro camera system was then used to collect images of the slices. According to the lung tissue injury score method, the four indexes of alveolar hyperemia (1 point), hemorrhage (1 point), neutrophil infiltration or aggregation in alveolar cavity or blood vessel wall (1 point), thickening of alveolar wall, and/or hyaline membrane formation (1 point) were used to evaluate the lung tissue injury score. The total score of lung injury was the sum of all the scores. 10 high-power visual fields (200 times) were observed in each section, and the average value was taken.

### 2.8. Enzyme-Linked Immune-Sorbent Assay (ELISA)

Lung tissues of rats in each group were taken to prepare homogenate, centrifuged at low temperature (4°C) at 10000 r/min for 10 min, and the supernatant was taken. The contents of IL-6, IL-1, hydroxyproline (HYP), GSH-Px, SOD, and MDA in lung tissues of rats were determined according to the manufacturer's protocols of the ELISA kit.

### 2.9. Immunohistochemical Staining

The lung tissues were fixed, paraffin‐embedded, sliced into serial sections, and dewaxed. After endogenous peroxidase was blocked using 3% H_2_O_2_, the sections were blotted with primary antibody anti-FN and anti-Col IV at 4°C overnight, followed by incubation with the secondary antibody. Finally, sections were stained with diaminobenzidine and then counterstained with hematoxylin. The slides were viewed using a light microscope (×400), and the optical density (OD) was analyzed by Image-Pro Plus Version 6.0 image analyzing system.

### 2.10. Real-Time Quantitative PCR (RT-qPCR)

Total RNA was isolated from the superior lobe of right lung tissues using an RNA extraction kit according to the manufacturer's instructions. The eligible RNA was then reverse transcribed into cDNA using a cDNA kit. After cDNA synthesis, mRNA expression levels of TGF*β*1, NOX4, PDGFR-*β*, and ROCK-1 were tested using the Ultra SYBR Mixture. Relative gene expression was calculated using the 2^−ΔΔCT^method and normalized against GAPDH. RT-qPCR reaction conditions were as follows: initial denaturation at 95°C for 10 minutes, followed by denaturation at 95°C for 10 minutes, annealing at 60°C for 10 minutes, and extension at 72°C of 10 minutes for 45 cycles. The CT value was recorded. The used primers were listed in Table 1.

### 2.11. Western Blot Analysis

For the determination of protein expression levels, proteins were extracted from lung tissue. Proteins were then separated by 10% SDS-PAGE and transferred onto PVDF membranes. The membranes were blotted with primary antibodies against TGF*β*1, NOX4, PDGFR-*β*, and ROCK-1; *ß*-actin was used as a loading control. The membranes were washed 3 times with TBST buffer for 10 min each time, followed by incubation with the secondary antibody. The polyvinylidene fluoride film was removed, and the film was obtained by the chemiluminescence method. The A value of the target band was analyzed using Quantity One gel imaging software, the ratio of the A value of the target band to the *ß*-actin band was considered as the relative expression of the target protein, and the number of repetitions was 3 times.

### 2.12. Statistical Analysis

The data were statistically analyzed using SPSS 20.0 software (IBM Corp.). All data results are tested by normal distribution first. After verification of a normal or nonnormal distribution by the Shapiro-Wilk test, a two-tailed Student's *t*-test and ANOVA of Tukey'**s ***post hoc* were performed to analyze the variables of normal distribution. When data was not normally distributed, it was log-transformed. In all the analyses, *P* < 0.05 was considered to indicate a statistically significant difference.

## 3. Results

### 3.1. Effect of Yifei Decoction Combined with MitoQ on Pulmonary Injuries of IPF Rats

**Figure 1:**
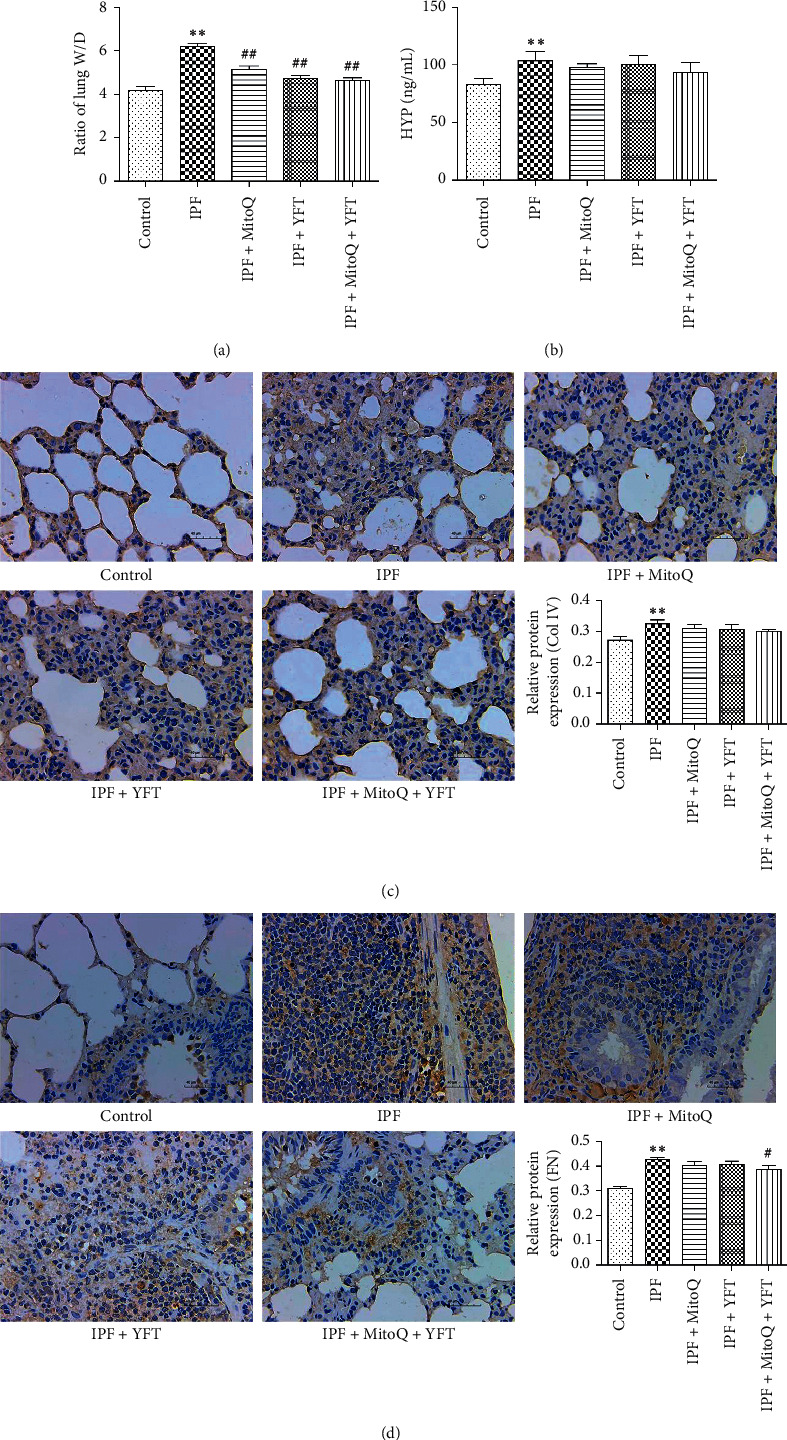
Effect of Yifei decoction combined with MitoQ on pulmonary injuries of IPF rats. (a) Ratio of lung W/D. (b) HYP content in lung tissue of rats in each group. (c) The expression of Col IV in each group. (d) The expression of FN in each group. All the sections were detected by immunohistochemical analysis. (c and d) Scale bar, 50 *μ*m. The data are expressed as the mean ± SD. Compared with the control group, ^*∗*^*P* < 0.05 and ^*∗∗*^*P* < 0.01; compared with the IPF group, ^#^*P* < 0.05 and ^##^*P* < 0.01.

Pulmonary fibrosis was characterized by collagen accumulation. HYP and Col IV were an index of collagen contents, and FN was the main noncollagen component. Analyses of HYP, Col IV, and FN contents were conducted to evaluate the effect of MitoQ + YFT. There was a simultaneous determination of lung tissue wet weight/dry weight ratio (W/D). Compared with the control group, the ratio of lung W/D and the contents of HYP were significantly increased in the IPF group (*P* < 0.01, Figures 1(a) and 1(b)), the expression of Col IV and FN was markedly increased in the IPF group, and positive cells are stained brownish yellow (*P* < 0.05, Figures 1(c) and 1(d)). Compared with the IPF group, the ratio of lung W/D was significantly decreased in the IPF + MitoQ, IPF + YFT, and IPF + MitoQ + YFT groups (*P* < 0.01, Figure 1(a)), and the expression of Col IV and FN could be inhibited in the group that were treated with MitoQ + YFT by immunohistochemistry testing (Figures 1(c) and 1(d)). These results indicated that MitoQ + YFT markedly ameliorated collagen accumulation in IPF rats.

### 3.2. Effect of Yifei Decoction Combined with MitoQ on Lung Tissue Pathology of IPF Rats

**Figure 2:**
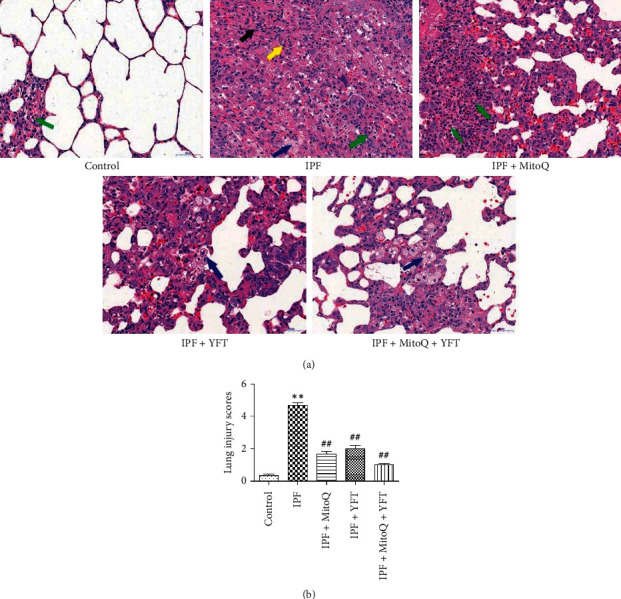
Effect of Yifei decoction combined with MitoQ on lung tissue pathology of IPF rats. (a) Representative micrographs of decalcified distal lung tissue paraffin sections stained with H&E (magnification, ×400). (b) Lung injury scores in each group. (a) Scale bar, 50 *μ*m; (**↑**) Neutrophils; (**↑**) Foam cells; (**↑**) Fibroblasts; (**↑**) Fibroblast. The data are expressed as mean ± SD. Compared with the control group, ^*∗*^*P* < 0.05 and ^*∗∗*^*P* < 0.01; compared with the IPF group, ^#^*P* < 0.05 and ^##^*P* < 0.01.

To identify the degree of pathological changes in lung tissue after treatment, sections of lung tissue were stained with H&E. No obvious lung tissue pathological changes were observed in rats in the control group by H&E staining. In the IPF group, the alveolar septum was significantly thickened, with more alveolar epithelial cell proliferation and inflammatory cell infiltration, including neutrophils, lymphocytes and foam cells, and fibrous tissue hyperplasia in the pulmonary interstitium. Fibroblasts with long oval nuclei and fibrous cells with long fusiform nuclei can be seen. In the IPF + MitoQ, IPF + YFT group, the bronchial structure at all levels of the lung was relatively normal, the bronchial ciliated epithelium was arranged neatly, the regional alveolar septum was thickened, and alveolar epithelial cell proliferation could be seen; different amounts of inflammatory cell infiltration could be seen in the pulmonary interstitium, mainly neutrophils and foam cells. In the IPF + MitoQ + YFT group, apparent alleviation of pulmonary alveolitis was observed. The pulmonary fibrosis zone was reduced largely, the alveolar structure was gradually healed, the alveolar septum was thinner, and the number of interstitial inflammatory cells was markedly reduced (Figure 2(a)). The results of quantitative evaluation of lung injury showed that the lung injury score of the IPF group was significantly higher than that of the control group, while that of the IPF + MitoQ group, the IPF + YFT group, and the IPF + MitoQ + YFT group was markedly lower than that of the IPF group (*P* < 0.01, Figure 2(b)). These data indicated that MitoQ + YFT improved the pathological changes of lung tissue in fibrotic rats with BLM-induced.

### 3.3. Effect of Yifei Decoction Combined with MitoQ on Inflammation and Oxidative Stress in the Lung Tissue of IPF Rats

**Figure 3:**
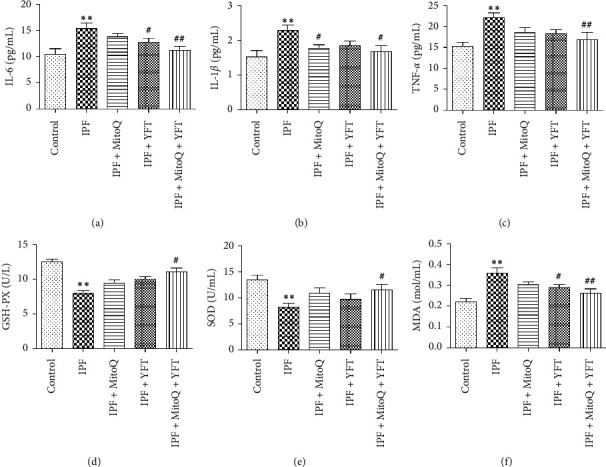
Effect of Yifei decoction combined with MitoQ on inflammation and oxidative stress in the lung tissue of IPF rats. (a) IL-6 levels in lung tissue. (b) IL-1*β* levels in lung tissue. (c) TNF-*α* levels in lung tissue. (d) GSH-Px activity. (e) SOD activity. (f) Lung tissue MDA content. The data are expressed as the mean ± SD. Compared with the control group, ^*∗*^*P* < 0.05 and ^*∗∗*^*P* < 0.01; compared with the IPF group, ^#^*P* < 0.05 and ^##^*P* < 0.01. IL, interleukin; TNF-*α*, tumor necrosis factor *α*; MDA, malonaldehyde; GSH-Px, glutathione peroxidase; SOD, superoxide dismutase.

Pulmonary fibrosis is closely related to oxidative stress and the release of a large number of inflammatory factors. The contents of inflammatory factors and oxidative stress indexes in lung tissue were analyzed to evaluate the anti-inflammatory and antioxidant activities of MitoQ + YFT. Compared with the control group, the levels of IL-6, IL-1*β*, TNF-*α*, and MDA were significantly increased in the IPF group (*P* < 0.01, Figures 3(a)–3(c) and 3(f)), whereas SOD and GSH-Px activities were significantly decreased in the IPF group (*P* < 0.01, Figures 3(d) and 3(e)). Compared with the IPF group, the levels of IL-6 and MDA were significantly decreased in the rats in the IPF + YFT and IPF + MitoQ + YFT groups (*P* < 0.05, Figures 3(a) and 3(f)), the levels of IL-1*β* were markedly decreased in the rats in the IPF + MitoQ and IPF + MitoQ + YFT groups (*P* < 0.05, Figure 3(b)), the levels of TNF-*α* were significantly decreased in the rats in the IPF + MitoQ + YFT group (*P* < 0.05, Figure 3(c)), and SOD and GSH-Px activities were significantly increased in the rats in the IPF + MitoQ + YFT group (*P* < 0.05; Figures 3(d) and 3(e)). These results indicated that IPF increased the levels of oxidative stress-related markers in lung tissue, whereas MitoQ and YFT reduce inflammation and oxidative stress in IPF rats.

### 3.4. Effect of Yifei Decoction Combined with MitoQ on TGF*β*1/NOX4 and PDGF/ROCK Signal Pathway in Lung Tissue of IPF Rats

**Figure 4:**
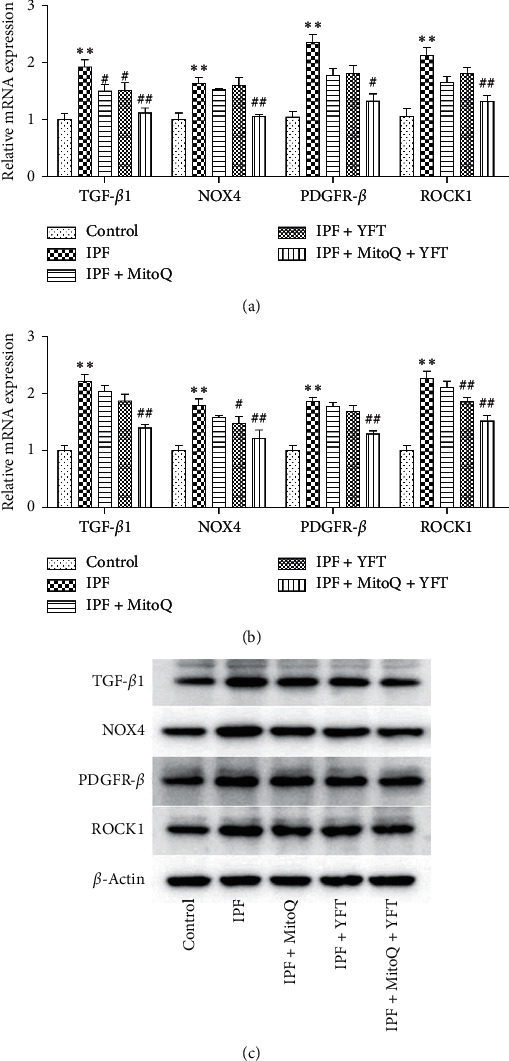
Effect of Yifei decoction combined with MitoQ on TGF*β*1/NOX4 and PDGF/ROCK signal pathway in lung tissue of IPF rats. (a) Relative mRNA expression of TGF*β*1, NOX4, PDGFR-*β*, and ROCK1. (b) Relative protein expression of TGF*β*1, NOX4, PDGFR-*β*, and ROCK1. (c) Protein band calculated as a ratio relative to *ß*-actin protein levels. The data are expressed as the mean ± SD. Compared with the control group, ^*∗*^*P* < 0.05 and ^*∗∗*^*P* < 0.01; compared with the IPF group, ^#^*P* < 0.05 and ^##^*P* < 0.01.

In order to determine the changes of TGF*β*1/NOX4 and PDGF/ROCK signal pathway in idiopathic pulmonary fibrosis after Yifei decoction combined with MitoQ intervention, we detected the expression levels of TGF*β*1, NOX4, PDGFR-*β*, and ROCK1. Compared with the control group, the mRNA and protein expression of TGF*β*1, NOX4, PDGFR-*β*, and ROCK1were significantly increased in the rats in the IPF group (*P* < 0.01, Figures 4(a)–4(c)). Compared with the IPF group, the mRNA expressions of TGF*β*1 in the IPF + MitoQ, IPF + YFT, and IPF + MitoQ + YFT groups were significantly decreased (*P* < 0.05; Figure 4(a)); the protein expression of TGF*β*1 in the IPF + MitoQ + YFT group was significantly decreased (*P* < 0.05; Figures 4(b) and 4(c)); the mRNA expressions of NOX4, PDGFR-*β*, and ROCK1 were markedly decreased in the rats in the IPF + MitoQ + YFT group (*P* < 0.05; Figure 4(a)); the protein expressions of NOX4 and ROCK1 in the IPF + YFT and IPF + MitoQ + YFT groups were significantly decreased (*P* < 0.05; Figures 4(b) and 4(c)); the protein expression of PDGFR-*β* was significantly decreased in the rats in the IPF + MitoQ + YFT group (*P* < 0.01, Figures 4(b) and 4(c)). These results indicate that IPF increased the mRNA and protein levels of related factors in the TGF*β*1/NOX4 and PDGF/ROCK pathways, while MitoQ and YFT reduced the mRNA and protein levels of related factors in this pathway in IPF rats.

## 4. Discussion

In the present study, we demonstrated that Yifei decoction combined with MitoQ not only delayed the pathological process of bleomycin-induced pulmonary fibrosis in rats but also ameliorated the lung injury of pulmonary fibrosis rats. Those might be achieved through reducing inflammatory infiltration of lung tissue, reducing collagen content, and inhibiting TGF*β*1/NOX4 and PDGF/ROCK signal pathways in fibrotic rats. These results showed that Yifei decoction combined with MitoQ provides a new method for the treatment of pulmonary fibrosis.

In animal experimental studies, bleomycin-induced pulmonary fibrosis models in rats or mice are generally used to study the pathogenesis and corresponding treatments. Bleomycin has been used as an antineoplastic drug, but it is now thought to cause dose-dependent interstitial pulmonary fibrosis [25]. Chen et al. [26] have reported that bleomycin can activate endoplasmic reticulum stress-related proteins *in vivo* and *in vitro*, resulting in the increase of lung fibroblasts. The deposition of extracellular matrix with extensive pulmonary septum is an important pathological feature of pulmonary fibrosis. Collagen is the main component of the extracellular matrix. HYP is a unique amino acid, which constitutes the collagen of the human body. Except for the fact that elastin contains a small amount of HYP (about 1%), almost all HYP exists in the form of collagen. Therefore, the content of HYP in tissue can directly reflect the degree of organ fibrosis [27]. When the level of W/D in lung tissue increased, the pulmonary edema was severe and a large number of inflammatory cells were infiltrated in the alveoli [28]. Col IV is the main component of the extracellular matrix. During fibrosis, a large number of interstitial components such as collagen can be synthesized and secreted [29]. FN increased in the early stage of fibrosis as a scaffold for collagen deposition [30]. Therefore, FN and Col IV are the direct indexes of collagen content. In our study, we used HYP, FN, and Col IV as an indicator of the degree of fibrosis. The results showed that Yifei decoction combined with MitoQ could significantly reduce the content of HYP, FN, and Col IV in lung tissue of rats with pulmonary fibrosis induced by bleomycin. In addition, IL-6, IL-1*β*, and TNF-*α* are proinflammatory cytokines that participate in many inflammatory responses of the lung, and studies have shown that the abnormal expression of IL-6, IL-1*β*, and TNF-*α* is related to the pathogenesis of respiratory diseases. Antioxidant enzymes such as GSH-Px and SOD constitute the antioxidant defense system of the body. Once there is an imbalance between oxidative damage and antioxidant damage, it will lead to the decrease of inflammation and immune function and the occurrence of biofilm lipid peroxidation, resulting in large area cell damage and fibrosis of tissues and organs. MDA is the end product of major lipid peroxidation of the cell membrane, which is produced under the action of lipid peroxidation and has cytotoxicity [31, 32]. Therefore, the determination of the changes of GSH-Px, SOD, and MDA in lung tissue can effectively predict the degree and trend of fibrosis. In this experiment, we found that Yifei decoction combined with MitoQ could significantly reduce the expression of IL-6, IL-1*β*, TNF-*α*, and MDA and increase the activity of GSH-Px and SOD in lung tissue of rats with pulmonary fibrosis induced by bleomycin. This may be one of the reasons for the improvement of lung function in rats with pulmonary fibrosis.

At present, the relationship between TGF*β*1/NOX4 and PDGF/ROCK signaling pathways is not very clear. In the study of lung diseases, NOX4/ROS can activate the RhoA/ROCK1 signal pathway, promote the migration of pulmonary fibroblasts and collagen synthesis, and aggravate pulmonary fibrosis [33]. In the study of renal disease, it was found that RhoA/ROCK1 is the upstream signal molecule of NOX4/ROS, and the activation of the RhoA/ROCK1 signal pathway can upregulate the expression of NOX4/ROS, promote the differentiation of renal myofibroblasts, and aggravate renal fibrosis [34]. Further review of the literature showed that both TGF*β*1 and AngII could upregulate the expression of NOX4, and GKT137831, a dual inhibitor of NOX1/4, could inhibit the production of ROS and hepatic fibrosis. It can be seen that NOX4 mediates the signal transduction of major hepatic fibrosis factors such as TGF*β*1 and plays an important role in the development of hepatic fibrosis [35]. PDGF can be divided into PDGFI and PDGFII, and it is expressed in a variety of cells. It can be activated by pulmonary monocytes and alveolar macrophages and can promote the division and proliferation of many kinds of cells [36]. PDGF can activate PDGFR and the complex Sos1 (guanine nucleotide exchange factor) by binding with Grb2 through its SH2 and SH3 regions, respectively. Sos-1 activates Ras, in turn leading to the activation of downstream Raf-1 and MAPK-ERK, and MAPK-ERK signal-activated gene transcription leads to downstream cell growth, differentiation, and migration. It participates in the neural signal transduction related to pulmonary fibrosis, including MAPK neural signal transduction pathway, PKC neural signal transduction pathway, TGF*β*1/Smad neural signal transduction pathway, and JAK-STAT neural signal transduction pathway. These signaling pathways are downstream proteins of a variety of cytokines (such as PDGF and TGF*β*1). These cytokines restrict and regulate each other, thus forming a complex network of cytokines in the lung, which participate in the formation of pulmonary fibrosis [37–39]. Therefore, the TGF*β*1/NOX4 and PDGF/ROCK pathways regulate each other in the process of pulmonary fibrosis. In this experiment, we confirmed that the expression of TGF*β*1, NOX4, PDGF, and ROCK increased significantly in the rat model of pulmonary fibrosis. After treatment with Yifei decoction combined with MitoQ, the expression of TGF*β*1, NOX4, PDGF, and ROCK in lung tissue decreased significantly. This may be another reason for the improvement of lung injury in rats with pulmonary fibrosis.

In conclusion, after the establishment of the bleomycin model, the contents of HYP, FN, Col IV, IL-6, IL-1*β*, TNF-*α*, and MDA, as well as the expression of TGF*β*1, NOX4, PDGF, and ROCK, in lung tissue of rats were significantly increased, and the lung tissue of rats was infiltrated by inflammation. These observations suggest that lung injury and activation of TGF*β*1/NOX4 and PDGF/ROCK signals do exist in the rat model of bleomycin-induced fibrosis. After treatment with Yifei decoction combined with MitoQ, the signal of TGF*β*1/NOX4 and PDGF/ROCK and the development of pulmonary fibrosis were inhibited. Therefore, Yifei decoction combined with MitoQ can inhibit the TGF*β*1/NOX4 and PDGF/ROCK signal pathway in fibrotic lung tissue. This study provides a basis for the study of traditional Chinese medicine compound prescription, provides a new method for the treatment of pulmonary fibrosis, and lays an experimental foundation for exploring new targets for the treatment of pulmonary fibrosis.

## Figures and Tables

**Table 1:** The primer sequences used in RT-qPCR for specific mRNA amplification.

Primer	Forward primer (5'-3')	Reverse primer (5'-3')
TGF-*β*1	ATGACATGAACCGACCCTTC	ACTTCCAACCCAGGTCCTTC
NOX4	GAAGGGGTTAAACACCTCTGC	ATGCTCTGCTTAAACACAATCCT
PDGFR-*β*	GTGCTCACCATCATCTCCCT	ACTCAATCACCTTCCATCGG
ROCK-1	TGCTGCTGGATAAATCTGGA	ATAACCATCGCCACCTTGAG
GAPDH	AGGAGCGAGACCCCACTAACA	AGGGGGGCTAAGCAGTTGGT

## Data Availability

The figure data used to support the findings of this study are included within the article.
